# Psychometric properties and gender invariance of the 8-item emotion regulation questionnaire (ERQ-8) among Chinese university students

**DOI:** 10.1371/journal.pone.0296035

**Published:** 2024-01-02

**Authors:** Mengyuan Zhao, Garry Kuan, Ke Zhou, Rabiu Muazu Musa, Anwar P. P. Abdul Majeed, Yee Cheng Kueh

**Affiliations:** 1 Exercise and Sports Science Programme, School of Health Sciences, Universiti Sains Malaysia, Kubang Kerian, Kelantan, Malaysia; 2 Sports Reform and Development Research Center of Henan University, School of Physical Education, Henan University, Kaifeng, China; 3 Centre for Fundamental and Continuing Education, Universiti Malaysia Terengganu, Kuala Nerus, Terengganu, Malaysia; 4 School of Robotics, XJTLU Entrepreneur College (Taicang), Xi’an Jiaotong-Liverpool University, Suzhou, China; 5 Biostatistics and Research Methodology Unit, School of Medical Sciences, Universiti Sains Malaysia, Kubang Kerian, Kelantan, Malaysia; Vellore Institute of Technology, INDIA

## Abstract

**Background:**

To assess emotion regulation strategies in a clear and direct manner, Emotion Regulation Questionnaire (ERQ) was developed based on the process model of emotion regulation. ERQ primarily assesses an individual’s propensity for reappraisal (a cognitive change in the individual’s psychological state in specific situations) and expressive suppression (a regulatory response where an individual alters their emotional response after the onset of an emotional reaction). Recent studies have suggested that the abbreviated 8-item version of the ERQ exhibits comparable model fit to the original version. The present study aimed to explore the psychometric properties and assess cross-gender invariance of the ERQ-8 in Chinese university students.

**Methods:**

University students from Jiangsu Province participated in this study. Participants completed self-report surveys assessing emotion regulation strategies. It was conducted from May 2022 to July 2022. The study employed confirmatory factor analysis (CFA) to assess the two-factor model of ERQ-8 and measurement invariance across male and female samples.

**Results:**

The mean age of 1534 participants was 19.83 years (SD = 1.54), and the majority were female (70.4%). The initial ERQ-10 model with ten items demonstrated good fit for all indicators, CFI (Comparative Fit index) = 0.967, TLI (Tucker-Lewis Index) = 0.957, RMSEA (Root Mean Square Error of Approximation) = 0.043, SRMR (Standardised Root Mean Square Residual) = 0.029. However, to assess the fit of the previously proposed ERQ-8 model, two items (Q1 and Q3) were excluded. The fit of the ERQ-8 model was further improved (CFI = 0.989, TLI = 0.984, RMSEA = 0.029, SRMR = 0.021). All item loadings exceeded or were equal to 0.573. Internal consistency analysis based on the ERQ-8 model revealed Cronbach’s alpha values of 0.840 for reappraisal and 0.745 for suppression, and corresponding composite reliability (CR) values of 0.846 and 0.747, respectively. Test-retest reliability, assessed using the intraclass correlation coefficient (ICC) (95% CI) within a one-week interval, ranged from 0.537 to 0.679. The correlation coefficient between the two factors was 0.084, significantly below 0.85, which suggested a low correlation between the two factors. The results of the invariance analysis across gender demonstrated that the values of ΔCFI and ΔTLI were both below 0.01. It was supported the gender invariance of the ERQ-8 among university students.

**Conclusion:**

The eight-item ERQ demonstrated validity and reliability in evaluating emotion regulation strategies, and measurement invariance was observed across gender among university students. The ERQ-8 may prove to be a practical and cost-effective tool, particularly in time-constrained situations.

## Introduction

Emotion regulation, a process utilized by individuals to manage, experience and express emotions, is acknowledged as a crucial mechanism underpinning mental and physical well-being [1]. As people utilise various strategies to manage both positive and negative emotions, the regulation of emotions becomes intricately linked to one’s physical and mental well-being, social connections, and emotional capabilities [1–3].

These strategies can be categorised into two types: antecedent-focused, which frequently seeks to reframe events positively, and reaction-focused, which aims to inhibit, conceal, or diminish emotional expression [4]. Building upon these two conscious or unconscious strategies that individuals employ in their daily lives, Gross & John [1] developed the Emotion Regulation Questionnaire (ERQ). Antecedent-focused strategies encompass cognitive reappraisal, which involves modifying one’s thoughts about situations that evoke emotions to alter the resulting emotional experience. Conversely, reaction-focused strategies involve expressive suppression, which entails inhibiting the outward display of emotions to regulate external emotional behaviour [1,4].

The ERQ has emerged as the most widely utilized tool for assessing emotion regulation strategies, with its extensive translation into multiple languages and successful validation and application [2,5–9]. These versions encompass various languages, such as Italian [2], Spanish [5], Turkish [6], Simplified Chinese [7], German [8], and Persian [9]. These studies have provided evidence for the validity and reliability of the translated versions of the ERQ. For example, in the Turkish version, the internal consistencies were 0.78 for the reappraisal and 0.73 for the suppression subscales [6]. Similarly, in the Persian version of the questionnaire, Cronbach’s alpha coefficients were reported as 0.76 for the reappraisal and 0.72 for the suppression subscales [9].

While the majority of ERQ versions have maintained the original model structure and demonstrated satisfactory fit across diverse cultures, validation studies conducted on non-student populations have revealed suboptimal or marginally adequate model fit for the 10-item ERQ. Hence, different versions of ERQ have been proposed. In a separate study by Spaapen et al. [10], the two-factor structure of the ERQ was tested on a community sample from two countries, resulting in a marginally adequate model fit. Nevertheless, by removing item Q3 from the scale, the model exhibited a good fit. Consequently, the authors proposed a shorter 9-item version of the ERQ (ERQ-9). Similarly, Rice et al. [11] arrived at a similar conclusion, the 9-item ERQ model with one item removed exhibited good fit. Brady et al. [12] found no significant difference in the fit statistics between the 9-item and 10-item ERQ models, indicating that both versions were acceptable. Building on these findings, Balzarotti [13] compared the original ERQ and ERQ-9 in both Italian community and student samples. In both cases, the original factor structure of the ERQ demonstrated poor fit. Although the model fit improved after removing item 3, the factor loading of item 1 showed a reduced value of 0.09 in both samples. In order to further improve the validation of the ERQ, abbreviated (8-item) version of the ERQ-8 was developed.

While the ERQ-8 may potentially serve as a more efficient instrument for assessing the emotion regulation strategy, the reliability and validity of the Chinese version of ERQ-8 have not yet been studied. In a recent cross-cultural comparative study [14], the ERQ-8 demonstrated good construct validity in the majority of 29 countries; however, the study’s sample did not include participants from the Chinese region. Moreover, research has observed variations in the use of emotion regulation strategies between genders [15]. Ensuring questionnaire equivalence across genders is essential as a preliminary step in measuring gender disparities. Therefore, it is essential to conduct measurement invariance tests on questionnaires for participants of different genders to enhance the value of these questionnaires. However, the measurement invariance of the Chinese version of ERQ-8 (ERQ-8-C) across different genders has not yet been validated.

In situations where time is limited, opting for a shorter version may be a suitable choice and could offer convenience in assessing emotional regulation strategies [13]. According to the findings from the aforementioned research, the ERQ-8 is the most concise version currently proposed, and it demonstrated satisfactory reliability and validity. Since the psychometric properties of the ERQ-8 have not been explored in the Chinese population, it is significant to conduct a psychological measurement assessment of the Chinese version of ERQ-8. Concurrently, ensuring measurement invariance becomes essential to facilitate valid comparisons across different groups [16]. The main objective of this study is to examine the psychometric properties of the ERQ-8 within the Chinese university student population, and assess its measurement invariance across different genders. In greater detail, in this study, (1) it conducted Confirmatory Factor Analysis (CFA) on a Chinese university student sample to compare the model fit of the original 10-item ERQ with the 8-item ERQ, thereby establishing the construct validity of ERQ-8; (2) following the determination of ERQ-8’s construct validity, the study evaluated its measurement invariance across male and female samples. Based on the content of this study, it anticipated that the satisfactory reliability and validity of ERQ-8 and the measurement invariance of different genders would be confirmed in the present study.

## Materials and methods

### Participants

Participants were recruited based on specific criteria, including being aged between 18 and 26, possessing Chinese nationality, and being native Chinese-speaking university students who demonstrated a strong ability to comprehend and respond to the questionnaire. The criteria for excluding questionnaire data included: patterned responses, obvious response bias (such as consistently selecting the same option), duplicate responses, and incorrect response to the general knowledge question (for instance, "From which direction does the sun rise?"). As all the content that needs to be analysed was set as mandatory, there was no missing data. In the study, 1660 questionnaires were received, 126 were excluded, and 1534 remained for analysis. A minimum sample size of 500 participants was recommended for CFA according to Hair et al. [17], and thus the study utilized a sample size of 1534, fulfilling this criterion.

### Measures

Apart from the content of the ERQ, the study also explored fundamental participant information such as age, gender, major, and academic year.

### Emotion regulation questionnaire

The original ERQ, developed by Gross and John [1], consisted of ten items measuring two dimensions: cognitive reappraisal (six items) and expressive suppression (four items). Participants rated their agreement with each item on a 7-point Likert-type scale, where "1" indicated complete disagreement and "7" indicated total agreement. The higher the score for each strategy, the more inclined one is to employ the corresponding emotion regulation strategy. For this study, the translation process was not conducted, since it utilized the ERQ-C, which was translated by Wang et al. [7] following a rigorous forward and backward translation process to ensure accuracy.

### Procedure

This study employed a cross-sectional research design and obtained approval from the Universiti Sains Malaysia (USM)’s Human Research Ethics Committee (USM/JEPeM/22040240) and was conducted in accordance with the guidelines of the International Declaration of Helsinki [18]. A convenience sampling method was utilized to recruit participants for questionnaire completion. Data collection was conducted exclusively online, from May 2022 to July 2022, with participants signed the online consent before beginning the study. The participant recruitment process involved using the Sojump (an electronic survey platform) to create an electronic questionnaire, generating a questionnaire link and QR code, producing posters, and displaying them. To maximize the study’s exposure, links were shared within Jiangsu Province, China, with various university student organizations’ WeChat groups, including class groups and student union groups. Participants could click on these links to participate in the survey. Moreover, after the initial response, participants were given the option to partake in the same research study again. If they expressed interest, they were added to a WeChat group, and after a seven-day interval (A week’s time is not so long that one’s emotional state would change, nor is it so short that one would clearly remember the content of their responses [19]), the link was reissued for further responses.

### Statistical analysis

The analysis of the data was performed utilising Mplus 8.3, no missing values in the data. Measurement data were presented as mean ± standard deviation, while count data were expressed as frequencies and percentages. The Mardia multivariate skewness and kurtosis tests of fit indicated that the presumption of multivariate normality was not satisfied (p<0.001). Consequently, an alternative estimator, MLM, which is robust to maximum likelihood, was employed in CFA and the testing of measurement invariance [20].

To evaluate the model fit, the following indicators were employed: χ^2^, df, χ^2^/df, Comparative Fit Index (CFI), Tucker-Lewis Index (TLI), Root Mean Square Error of Approximation (RMSEA) with its 90% confidence interval (90% C.I.), and root mean square residual (SRMR). Each indicator was required to meet the following criteria: χ^2^/df < 3, CFI > 0.90, TLI > 0.90, RMSEA < 0.08, and SRMR < 0.05 [21].

Reliability was checked using SPSS 26.0. Cronbach’s alpha, exceeding 0.6, confirmed acceptable reliability [22]. Yet, it’s worth noting that this measure might undervalue reliability due to error correlation. To address this, composite reliability (CR) was computed for a supplementary assessment, with CR’s critical value set at a minimum of 0.60 [23]. Discriminant validity was assessed using the correlation coefficient. A coefficient below 0.85 indicated differentiation among individual items of the questionnaire [24]. Furthermore, a threshold of 0.40 or above was set for factor loadings. Items with factor loadings below 0.40 were considered questionable [25]. Additionally, the ICC was used in a test-retest reliability analysis over one week. ICC values were categorised as follows: less than 0.4 (poor), between 0.4 and 0.59 (fair), between 0.6 and 0.74 (good), and 0.75 or above (excellent) [26].

Finally, a multi-group CFA was conducted to examine the invariance of the ERQ-8 across gender in the university student population. Measurement invariance encompassed four aspects: (1) Configural invariance, (2) Weak invariance, (3) Strong invariance and (4) Strict invariance. In this study, ΔCFI and ΔTLI were used to evaluate invariance, and the invariance model was deemed acceptable if ΔCFI ≤ 0.01 and ΔTLI ≤ 0.01 were satisfied [27,28].

## Results

A total of 1534 university students successfully completed the ERQ as part of this study. The average age of the participants was 19.83 years. Among the respondents, female students constituted the majority of the study population. With regard to the distribution of majors, the overwhelming majority of participants were pursuing degrees in Medicine. Regarding the distribution of participants across academic years, freshmen and sophomores accounted for the largest proportion. Given that the primary distribution locations for the questionnaire were areas with a higher concentration of female students and individuals majoring in medicine, along with a larger population of lower-year university students on campus, the resulting data trends were expected. For more comprehensive information, please refer to Table 1.

**Table 1:** The demographic characteristics (*n* = 1534).

Characteristics	Frequency	Percentage	Mean ±SD
**Gender**			
Male	454	29.60%	
Female	1080	70.40%	
**Age (years)**			19.83±1.54
**Major**			
Science and Engineering	36	2.35%	
Medicine	1311	85.46%	
Agriculture	3	0.19%	
Economics	32	2.09%	
Management	38	2.48%	
Law	20	1.30%	
Pedagogy	87	5.67%	
Art	7	0.46%	
**Class**			
Freshman	718	46.81%	
Sophomore	583	38.01%	
Junior	132	8.60%	
Senior	63	4.10%	
Others	38	2.48%	

### Measurement model for the ERQ

This study evaluated the model fit using χ^2^/df, CFI, TLI, RMSEA, and SRMR. Typically, a smaller χ^2^/df value indicates a better fit of the model in the data. The closer the CFI and TLI values are to 1, the better the model fits. The closer the RMSEA and SRMR values are to 0, the better the model fits [21].

The goodness-of-fit indices obtained from Model-1-10 indicated a satisfactory fit, with the exception of a slightly elevated χ^2^/df value. Nonetheless, all other metrics exceeded the recommended thresholds.

Building upon Model-1-10, two items (Q1 and Q3) were removed from the reappraisal factor to create Model-2-8. The revised hypothetical measurement model, ERQ-8, consisted of 8 items distributed across the two dimensions. Remarkably, all the fit indices for this model demonstrated an improvement over Model-1-10, suggesting a better fit to the data.

In terms of model fitting indicators, the ERQ model with ten items was already satisfactory. However, the model fit for the eight-item ERQ had been refined even further. To be specific, the χ^2^/df ratio had seen a notable decrease of 1.63, accompanied by respective improvements of 0.022 and 0.027 in CFI and TLI. Additionally, both RMSEA and SRMR had registered decreases of 0.014 and 0.008 respectively. Additional information can be found in Table 2.

**Table 2:** A summary of the models’ fit indices.

Path Models	χ^2^	df	χ^2^/df	CFI	TLI	RMSEA (90% C.I.)	SRMR
Model 1–10	132.219	34	3.89	0.967	0.957	0.043 (0.036–0.051)	0.029
Model 2–8	43.010	19	2.26	0.989	0.984	0.029 (0.017–0.040)	0.021

TLI = Tucker-Lewis index; CFI = comparative fit indices; RMSEA = root mean square error of approximation; SRMR = standardized root mean square.

For both Model-1-10 and Model 2–8, Notably, all factor loadings surpassed the critical value of 0.4. The factor loading signifies the degree of correlation between an observed variable and its corresponding latent factor [29]. In this study, all observed variables in both models had factor loadings exceeding 0.4, indicating a relatively robust association between all observed variables and their respective latent factor. They had the range from 0.553 to 0.840 in Model-1-10, and all factor loadings ranged from 0.573 to 0.875 in Model 2–8. Table 3 shows the Standardised factor loadings.

**Table 3:** Standardised factor loadings for Model-1-10 and Model-2-8 of the ERQ.

Factors and Items	Factor Loadings	
	Model 1–10	Model 2–8
**Reappraisal**		
Q1	0.553	-
Q3	0.620	-
Q5	0.643	0.624
Q7	0.813	0.795
Q8	0.840	0.875
Q10	0.736	0.735
**Suppression**		
Q2	0.573	0.573
Q4	0.590	0.590
Q6	0.757	0.757
Q9	0.681	0.681

### Reliability and validity

Cronbach’s alpha values were computed for the Reappraisal and Suppression factors based on the ERQ-8 measurement model, yielding values of 0.840 and 0.745, respectively. Additionally, the CR estimates for Reappraisal (0.846) and Suppression (0.747) exceeded the acceptable threshold. According to Cronbach’s alpha and CR values, scores above 0.7 are generally considered good, and above 0.8 are excellent [22,23]. In this study, the factors of ERQ-8 demonstrated reliability at a "good" level or higher.

The correlation coefficient between the Reappraisal and Suppression factors was 0.084, which was highly significant. This correlation coefficient was significantly below the recommended threshold of 0.85. This indicated a relatively low correlation between the different factors, suggesting that the two factors of ERQ-8 possessed relatively independent measurement contents, without being excessively influenced by the other factor, indicating good discriminant validity of the ERQ-8. The detailed values of CR, Cronbach’s alpha, and correlation coefficient can be found in Table 4.

**Table 4:** Cronbach’s alpha, Composite reliability, and the factor correlation of the ERQ-8.

Variables	Cronbach’s alpha	Composite reliability	1	2
1. Reappraisal	0.840	0.846	1	0.084**
2. Suppression	0.745	0.747	-	1

**Correlation is significant at the 0.01 level (one-tailed).

Moreover, to assess the test-retest reliability, 65 participants filled out both pre and post questionnaires after a week of completing the first answers. 11 responses were discarded due to ineligibility, resulting in 54 valid questionnaires (14 males and 40 females). The test-retest reliability of each item, as measured by the ICC, ranged from 0.537 to 0.679. Based on the rating interval, the ICC values falling within the range of 0.4 to 0.59 indicate a level of fair retest reliability, while those within the range of 0.6 to 0.74 signify a level of good retest reliability. In the present study, the results demonstrated that each item exhibited test-retest reliability at or above the "fair" threshold. The results of the test-retest reliability for each item are presented in Table 5.

**Table 5:** Test–retest reliabilities of ERQ-C-8.

Item	Test 1	Test 2	ICC (95% CI)
***n* = 54**	**Mean ± SD**	**Mean ± SD**	
**Q2**	3.65 ± 1.01	3.80 ± 1.12	0.587
**Q4**	3.65 ± 1.15	3.72 ± 1.07	0.679
**Q5**	4.54 ± 0.93	4.54 ± 0.97	0.537
**Q6**	3.61 ± 1.20	3.61 ± 1.17	0.556
**Q7**	4.37 ± 1.02	4.15 ± 1.04	0.585
**Q8**	4.61 ± 0.79	4.56 ± 0.82	0.542
**Q9**	3.70 ± 1.11	3.76 ± 1.03	0.619
**Q10**	4.43 ± 0.92	4.39 ± 0.79	0.557

Notes: CI = Confidence Interval, ICC = Intraclass correlation coefficient.

### Measurement invariance

Multiple confirmatory factor analyses were conducted to examine the gender invariance of the ERQ-8 scale. Four successive models were developed: configural invariance, metric invariance, scalar invariance, and strict invariance. The findings are presented in Table 6. The CFI and TLI for all four models, as well as the separate models for males and females, exceeded the threshold of 0.90, indicating satisfactory model fit. Additionally, RMSEA was below 0.08, meeting the recommended criteria for model fit.

**Table 6:** ERQ-8 baseline model fit results and tests of invariance.

Models	χ^2^	df	χ^2^/ df	CFI	TLI	RMSEA (90%C.I.)	ΔCFI	ΔTLI
**Male**	21.788	19	1.15	0.996	0.994	0.018 (0.000–0.046)	-	-
**Female**	46.867	19	2.47	0.982	0.974	0.037 (0.024–0.050)	-	-
**Configural**	68.203	38	1.79	0.987	0.980	0.032 (0.019–0.044)	-	-
**Metric**	71.831	44	1.63	0.988	0.984	0.029 (0.016–0.040)	0.001	0.004
**Scalar**	82.490	50	1.65	0.986	0.984	0.029 (0.017–0.040)	-0.002	0
**Strict**	87.236	58	1.50	0.987	0.987	0.026 (0.013–0.036)	0.001	0.003

Through an in-depth analysis, it was observed that the changes in CFI and TLI between the metric and configural models fell within acceptable limits (ΔCFI = 0.001, ΔTLI = 0.004). This suggests that the measurement of factor scores remained invariant across genders, indicating that the questionnaire items used to assess factor loadings were interpreted similarly by both males and females. The scalar model, which maintains constant factor loadings and structural covariances, demonstrated a good fit to the data. Comparing the scalar model (more restricted) to the metric model (less restricted), the changes in CFI and TLI were minimal (ΔCFI = -0.002, ΔTLI = 0). This implies that factor loadings, as well as common covariance and variance, were consistent between genders. Despite imposing additional conditional restrictions, the strict model also displayed a favourable fit. The changes in CFI and TLI, compared to the scalar model, were all within acceptable limits (ΔCFI = 0.001, ΔTLI = 0.003). These results indicated that the relationship between the two variables of the ERQ scale remained consistent across gender, with no significant differences observed between male and female samples.

## Discussion

The ERQ is a widely adopted instrument utilized for assessing emotion regulation strategies. The ERQ-8 represents a more concise rendition of the ERQ that has recently been introduced. The present study examined the psychometric properties of the ERQ-8, as well as assessed its measurement invariance. To the best of our knowledge, this constituted the inaugural examination of the structural validity and measurement invariance of the Chinese version of the ERQ-8. The findings substantiated the satisfactory reliability and validity of the ERQ-8-C within the university student population, as well as its gender invariance across male and female samples, thus realizing the objective of this study.

### Construct validity

The results from the CFA indicated that both the 10-item ERQ and the 8-item ERQ exhibited sound structural validity. Firstly, the original structural sample data of the 10-item ERQ demonstrated a good fit, which aligned with findings reported in other studies of the Chinese version of ERQ-10 [30,31]. The 8-item ERQ, proposed by Balzarotti [13], involves the removal of items 1 and 3, both of which pertain to the reappraisal factor. Item 1 is considered to assess positive emotion regulation, while item 3 is regarded as measuring negative emotion regulation. The exclusion of item 1 and item 3 may be justified, as item 7 and 10 within the reappraisal subscale also capture closely related facets of the underlying construct. Removing these two similar items to form a shorter scale could potentially alleviate redundancy within the reappraisal factor [13]. In the present study, it reconstructed the ERQ-8 model through the removal of item 1 and items 3. The results indicated superior fit of the 8-item ERQ in the student sample compared to the 10-item ERQ. The factor loadings of each item exhibited satisfactory measurement properties. These findings furnished preliminary evidence for the validity of the 8-item Chinese version of the ERQ. Consequently, the ERQ-8 was employed for subsequent analyses.

### Reliability

Based on Cronbach’s alpha, CR coefficient, and ICC value, ERQ-8 demonstrated satisfactory reliability. In the reliability analysis of ERQ-8, both the Cronbach’s alpha and CR coefficients for different factors were ≥ 0.745, indicating a satisfactory level of internal consistency. Coupled with the results of the aforementioned CFA, it obtained favourable evidence for the internal consistency reliability and structural validity of ERQ-8, which align with findings from previous similar studies [30,32,33].

Additionally, in this study, it assessed the test-retest reliability of the ERQ-8, maintaining a one-week gap between the two sets of questionnaire responses. The chosen time interval aligns with that employed in prior research concerning test-retest reliability assessments [34,35]. The rationale for choosing a one-week interval for reliability analysis was that this period was short enough to ensure that the emotional state of the participants did not undergo significant changes, yet long enough to avoid the effects of test familiarity (i.e., participants performing better in the second test simply because they were familiar with the questionnaire content). The results of the test-retest reliability yielded an ICC value of ≥ 0.537, suggesting an above fair level of consistency between repeated measurements under similar conditions. This indicated that the differences in responses when using the same questionnaire at different time points are primarily due to true individual differences, rather than measurement error. The test-retest reliability in this study is consistent with prior similar research [2,36], indicating good test-retest reliability. These results supported the use of this abbreviated version of the ERQ measurement tool for assessing the emotion regulation strategies employed by Chinese university students.

### Measurement invariance

The assessment of measurement invariance analysis demonstrated that the two-factor model was valid and equivalent across male and female samples. This aligns with prior research findings, as evidenced by Zhang & Bian [32]. Multiple confirmatory factor analyses were employed to assess the degree of invariance exhibited by the ERQ-8 across various levels: configural invariance, metric invariance, scalar invariance, and strict invariance. The findings consistently provided support for these levels of invariance, indicating that the ERQ-8 maintained its measurement properties regardless of gender. Through the progressive imposition of restrictive conditions, the model demonstrated negligible changes, thereby preserving its primary psychometric characteristics. This signifies the stability of the ERQ-8 across different gender groups, affirming its applicability in assessing individuals’ emotional regulation tendencies. Configural invariance asserts that the ERQ-8 captures comparable psychological structures within diverse gender groups, as evidenced by the equivalence of factor quantities and patterns across these groups [37].

In the configural invariance model, the estimation of factor loadings, intercepts of observed variables, and other parameters was conducted without assuming any constancy across gender samples. Multiple sets of CFA were employed to investigate whether the latent variables exhibited the same structural patterns, encompassing the number of factors and the hierarchical arrangement of topics and factors. This established a foundational model for subsequent model nesting. The results consistently supported the configural invariance, indicating that the baseline model accurately captured the underlying structure across gender groups. From there, the weak invariance model was tested, wherein the measurement weights were set to be constant, and the corresponding factor loadings were constrained to be equal across groups. The findings indicated that the ERQ-8 demonstrated reciprocal relationships between each item and its corresponding latent variable in both male and female groups. Furthermore, it displayed equivalent units, thereby representing the same meaning across various dimensions of emotion regulation strategy scores between genders. As a result, direct comparisons and interpretations of ERQ-8 scores between males and females were valid. Subsequently, the strong invariance model was tested by introducing additional constraints to Metric model, particularly by setting the structural covariances equal. Although the fit of the model slightly diminished with the imposition of these extra restrictions, the assumption of equivalence could be considered reasonable. Consequently, the strong invariance assumption was not rejected in this study [38]. Finally, the strict invariance model added further restrictions to scalar model, specifically by setting the measurement residuals constant and equalizing the error variances. This indicated that the magnitude of measurement error attributed to random factors was identical across genders.

This study served as a complement to the investigation of the psychometric properties of the ERQ-8. In recent years, the ERQ has emerged as one of the most commonly employed self-report instruments for assessing emotion regulation strategies. Consequently, it is meaningful to comprehend and validate a briefer iteration of the ERQ that appears to possess superior psychometric properties. The ERQ-8 offers a succinct and simplified measure, which may prove to be more practical in assessment, research settings and large-scale training, particularly when time is constrained. Possibly, it may alleviate the testing burden on participants and reduce the costs associated with data collection and processing. Researchers could potentially conduct experiments or investigations more efficiently, as employing the 8-item ERQ could result in time and resource savings. This affords them the ability to garner a greater volume of data within the same timeframe, thereby fostering progress in research endeavours.

### Limitations

The present study necessitates consideration of several constraints and further avenues of inquiry. Firstly, while the dataset employed in this study substantially surpasses the minimum requirement for CFA, the recruitment of participants was conducted through a convenience sampling method, encompassing only a select demographic of university students from a limited number of regions. Consequently, this constrains the generalizability of the findings to the broader population of Chinese university students. To enhance the applicability and substantiate the robustness of the study’s findings, future research may benefit from employing a more diversified sample, potentially encompassing a broader spectrum of regional representation. Secondly, this study adopted a cross-sectional design, which did not account for potential temporal variations in emotion regulation strategies. For instance, states of psychological well-being may engender disparities in the utilization of emotion regulation strategies [39]. Thus, it is imperative to conduct longitudinal investigations to ascertain the measurement invariance of the ERQ-8-C over time. Thirdly, this study primarily scrutinized the content of construct validity of the ERQ-8, and there exists a gamut of additional psychometric properties warranting further validation, such as concurrent validity. Future research endeavours should contemplate a more comprehensive exploration of various facets of the psychometric properties of the ERQ-8. Fourthly, given the satisfactory outcomes of the research within the university student sample, the psychometric properties and measurement invariance of ERQ-8-C across diverse groups could be examined in future studies.

### Conclusion

In conclusion, this study investigated the psychometric properties of ERQ-8-C in a sample of Chinese university students. The abbreviated ERQ-8 provided a valid and reliable measure of emotion regulation strategies for this sample. The 8-item ERQ exhibited good model fit and measurement invariance across gender. The concise nature of the ERQ-8 may render it a practical and cost-effective instrument, particularly in scenarios where time limitations are a consideration.

## Supporting information

S1 File ERQ-C data. (XLS)

## References

[1] Gross JJ, John OP. Individual differences in two emotion regulation processes: Implications for affect, relationships and well-being. *Journal of Personality and Social Psychology*. 2003; 85(2): 348–362. doi: 10.1037/0022-3514.85.2.348 12916575

[2] Balzarotti S, John OP, Gross JJ. An Italian adaptation of the Emotion Regulation Questionnaire. *European Journal of Psychological Assessment*. 2010; 26(March):61–67. 10.1027/1015-5759/a000009.

[3] Gullone E, Taffe J. The Emotion Regulation Questionnaire for Children and Adolescents (ERQ-CA): A psychometric evaluation. *Psychological Assessment*. 2012; 24(2):409–417. doi: 10.1037/a0025777 22023559

[4] Gross J J. Antecedent- and response-focused emotion regulation: Divergent consequences for experience, expression, and physiology. *Journal of Personality and Social Psychology*. 1998; 74(1):224–237. doi: 10.1037//0022-3514.74.1.224 9457784

[5] Cabello R, Salguero JM, Fernández-Berrocal P, Gross JJ. A Spanish adaptation of the emotion regulation questionnaire. *European Journal of Psychological Assessment*. 2013; 29(4):234–240. 10.1027/1015-5759/a000150.

[6] Eldeleklioğlu J, Eroğlu Y. A Turkish adaptation of the emotion regulation questionnaire. *Journal of Human Sciences*. 2015; 12(1):1157–1168. 10.14687/ijhs.v12i1.3144.

[7] Wang L, Liu HC, Li ZQ, Du W. Reliability and validity of emotion regulation questionnaire Chinese revised version. *Chinese Journal of Health Psychology*. 2007; 15(6):503–505.

[8] Abler B, Kessler H. Emotion regulation questionnaire—Eine deutschsprachige Fassung des ERQ von Gross und John [Emotion regulation questionnaire—A German version of the ERQ by Gross and John]. *Diagnostica*. 2009; 55(3):144–152. 10.1026/0012-1924.55.3.144.

[9] Foroughi AA, Parvizifard A, Sadeghi K, Parsa Moghadam A. Psychometric properties of the Persian version of the Emotion Regulation Questionnaire. *Trends in Psychiatry and Psychotherapy*. 2021; 43(2):101–107. doi: 10.47626/2237-6089-2018-0106 34043902 PMC8317545

[10] Spaapen DL, Waters F, Brummer L, Stopa L, Bucks RS. The emotion regulation questionnaire: Validation of the ERQ-9 in two community samples. *Psychological Assessment*. 2014; 26(1):46–54. doi: 10.1037/a0034474 24059476

[11] Rice SM, Treeby MS, Gersh E, Ogrodniczuk JS, Kealy D. The emotion regulation questionnaire: ERQ-9 factor structure and measurement invariance in Australian and Canadian community samples. TPM Testing, *Psychometrics*, *Methodology in Applied Psychology*. 2018; 25(3):369–378. 10.4473/TPM25.3.3.

[12] Brady B, Kneebone II, Bailey PE. Validation of the Emotion Regulation Questionnaire in older community‐dwelling adults. *British Journal of Clinical Psychology*. 2019; 58(1):110–122. doi: 10.1111/bjc.12203 30151834

[13] Balzarotti S. The emotion regulation questionnaire: factor structure and measurement invariance in an Italian sample of community dwelling adults. *Current Psychology*. 2019; 40(10):4918–4929. 10.1007/s12144-019-00426-3.

[14] Burghart M, Sahm AH, Mier D. Investigating measurement invariance of the Emotion Regulation Questionnaire-8 (ERQ-8) across 29 countries. *Current Psychology*. 2023; 1–7. doi: 10.1007/s12144-022-04220-6 36627950 PMC9817443

[15] Seixas R, Pignault A, Houssemand C. Emotion regulation questionnaire-adapted and individual differences in emotion regulation. *Europe’s Journal of Psychology*. 2021; 17(1):70–84. doi: 10.5964/ejop.2755 33737975 PMC7957848

[16] Putnick DL, Bornstein MH. Measurement invariance conventions and reporting: The state of the art and future directions for psychological research. *Developmental Review*. 2016; 41(September):71–90. doi: 10.1016/j.dr.2016.06.004 27942093 PMC5145197

[17] Hair JF, Black WC, Babin BJ, Anderson RE, Tatham R. *Multivariate Data Analysis*. Upper Saddle River, NJ, USA: Pearson Prentice Hall. 2006.

[18] World Medical Association. World Medical Association Declaration of Helsinki: Ethical Principles for Medical Research Involving Human Subjects. *JAMA*. 2013; 310(20):2191–2194. doi: 10.1001/jama.2013.281053 24141714

[19] Wang Q. Gender and emotion in everyday event memory. *Memory*. 2013; 21(4):503–511. doi: 10.1080/09658211.2012.743568 23190136

[20] Muthe n LK, Muthe n BO. Mplus: The comprehensive modeling program for applied researchers: User’s guide: Muthe´n & Muthe´n; 1998.

[21] Wu ML. *Structural equation model*: *AMOS operation and application*. Chongqing: Chongqing University Press. 2011.

[22] Taber KS. The use of cronbach’s alpha when developing and reporting research instruments in science education. *Research in Science Education*. 2018; 48(6):1273–1296. 10.1007/s11165-016-9602-2.

[23] Fornell C, Larcker DF. Evaluating structural equation models with unobservable variables and measurement error: Algebra and statistics. *Journal of Marketing Research*. 1981; 18(1):39–50. 10.1177/002224378101800313.

[24] Kline RB. *Principles and Practice of Structural Equation Modelling*. New York, NY: Guilford Publications. 2015.

[25] DeVon HA, Block ME, Moyle‐Wright P, Ernst DM, Hayden SJ, Lazzara DJ, et al. A psychometric toolbox for testing validity and reliability. *Journal of Nursing Scholarship*. 2007; 39(2):155–164. doi: 10.1111/j.1547-5069.2007.00161.x 17535316

[26] Mason J, Classen S, Wersal J, Sisiopiku V. Construct validity and test–retest reliability of the automated vehicle user perception survey. *Frontiers in Psychology*. 2021; 12(January):626791. doi: 10.3389/fpsyg.2021.626791 33569031 PMC7868437

[27] Cheung GW, Rensvold RB. Evaluating goodness-of-fit indexes for testing measurement invariance. *Structural Equation Modelling*: *A Multidisciplinary Journal*. 2002; 9(2):233–255. 10.1207/S15328007SEM0902_5.

[28] Chen FF. Sensitivity of goodness of fit indexes to lack of measurement invariance. Structural *Equation Modelling*: *A Multidisciplinary Journal*. 2007; 14(3):464–504. 10.1080/10705510701301834.

[29] Shrestha N. Factor analysis as a tool for survey analysis. *American Journal of Applied Mathematics and Statistics*. 2021; 9(1):4–11. 10.12691/ajams-9-1-2.

[30] Gong J, Wang MC, Zhang X, Zeng H, Yang W. The Emotion Regulation Questionnaire for Children and Adolescents (ERQ-CA): Factor structure and measurement invariance in a Chinese student samples. *Journal of Personality Assessment*. 2022; 104(6):774–783. doi: 10.1080/00223891.2021.2014506 34962841

[31] Wang D, Yuan B, Han H, Wang C. Validity and reliability of emotion regulation questionnaire (ERQ) in Chinese rural-to-urban migrant adolescents and young adults. *Current Psychology*. 2022; 41:2346–2353. 10.1007/s12144-020-00754-9.

[32] Zhang Y, Bian Y. Emotion regulation questionnaire for cross-gender measurement invariance in Chinese university students. *Frontiers in Psychology*. 2020; 11:569438. doi: 10.3389/fpsyg.2020.569438 33250813 PMC7673430

[33] Chen W, Zhang G, Tian X, Wang L. Psychometric properties and measurement invariance of the emotion regulation questionnaire in Chinese left-behind children. Current Psychology. 2023; 42(11):8833–8843. 10.1007/s12144-021-02155-y.

[34] Vasheghani-Farahani A, Tahmasbi M, Asheri H, Ashraf H, Nedjat S, Kordi R. The Persian, last 7-day, long form of the International Physical Activity Questionnaire: translation and validation study. *Asian journal of sports medicine*. 2011; 2(2):106–116. doi: 10.5812/asjsm.34781 22375226 PMC3289200

[35] Arifin N, Osman NAA, Abas WABW. Intrarater test-retest reliability of static and dynamic stability indexes measurement using the Biodex Stability System during unilateral stance. *Journal of applied biomechanics*. 2014; 30(2):300–304. 10.1123/jab.2013-0130. 23878204

[36] Liu W, Chen L, Tu X. Chinese adaptation of Emotion Regulation Questionnaire for Children and Adolescents (ERQ‐CCA): A psychometric evaluation in Chinese children. *International Journal of Psychology*. 2017; 52(5):398–405. doi: 10.1002/ijop.12233 26611865

[37] Han K, Colarelli SM, Weed NC. Methodological and statistical advances in the consideration of cultural diversity in assessment: A critical review of group classification and measurement invariance testing. *Psychological Assessment*. 2019; 31(12):1481. doi: 10.1037/pas0000731 31763873

[38] Vandenberg RJ, Lance CE. A review and synthesis of the measurement invariance literature: Suggestions, practices, and recommendations for organizational research. *Organizational Research Methods*. 2000; 3(1):4–70. 10.1177/109442810031002.

[39] Rozgonjuk D, Elhai JD. Emotion regulation in relation to smartphone use: Process smartphone use mediates the association between expressive suppression and problematic smartphone use. *Current Psychology*. 2021; 40:3246–55. 10.1007/s12144-019-00271-4.

